# Impact of Hospital Hierarchy on Nurses’ Attitudes Toward Artificial Intelligence: Mediating Roles of Artificial Intelligence Literacy and Anxiety

**DOI:** 10.1155/jonm/5593996

**Published:** 2026-05-22

**Authors:** Ding Xu, Yimeng Zheng, Yongtian Yin, Cuixia Lin, Linlin Yang, Beibei Liang, Jielong Du

**Affiliations:** ^1^ School of Nursing, Shandong University of Traditional Chinese Medicine, Jinan, Shandong, China, sdutcm.edu.cn; ^2^ School of Health, Shandong University of Traditional Chinese Medicine, Jinan, Shandong, China, sdutcm.edu.cn; ^3^ Jinan Maternity and Child Care Hospital Affiliated to Shandong First Medical University, Jinan, Shandong, China

**Keywords:** artificial intelligence anxiety, artificial intelligence literacy, attitude toward the use of artificial intelligence, hospital hierarchy, mediation effect

## Abstract

**Background:**

With the rapid development of science and technology, the application of artificial intelligence in the field of healthcare is becoming increasingly widespread. As the executors and responsible persons of nursing work, nurses’ understanding and attitude toward AI technology determine whether AI can be deeply integrated and successfully applied in the field of nursing.

**Aims:**

To investigate nurses’ negative and positive attitudes toward the use of artificial intelligence and influencing factors, explore the mediating effect of artificial intelligence literacy and anxiety between hospital hierarchy differences and negative and positive attitudes toward the use of artificial intelligence, and provide basis for improving nurses’ attitudes toward the use of artificial intelligence.

**Methods:**

In November 2025, the convenience sample of 436 nurses from different hospitals in Shandong Province was surveyed. Data were collected using the general information questionnaire, the attitude scale toward the use of artificial intelligence technologies in nursing, the artificial intelligence anxiety scale, and the artificial intelligence literacy scale. Multiple linear regression analyzed the influencing factors of nurses’ negative and positive attitudes toward the use of artificial intelligence. Mediation analyses explored the mediating effect of artificial intelligence literacy and anxiety between the hospital hierarchy differences of nurses and their negative and positive attitudes toward the use of artificial intelligence.

**Results:**

The score of negative attitude was 14.55 ± 6.63, and the score of positive attitude was 38.21 ± 3.87. Artificial intelligence literacy and anxiety partially mediated the relationship between hospital hierarchy differences and the negative and positive attitudes toward the use of artificial intelligence, with the total mediating effects being 3.067 and −1.011, respectively.

**Conclusion:**

Hospital hierarchy differences could directly positively predict the negative and positive attitudes toward the use of artificial intelligence and could also indirectly positively predict the negative attitude toward the use of artificial intelligence through mediation by artificial intelligence literacy and anxiety and negatively predict the positive attitude toward the use of artificial intelligence.

**Implications for Nursing Management:**

Providing personalized artificial intelligence training based on the needs of hospitals could improve nurses’ attitudes toward the use of artificial intelligence, increase their artificial intelligence literacy, and reduce their anxiety.

## 1. Introduction

With the rapid development of science and technology, the application of artificial intelligence (AI) in the field of healthcare is becoming increasingly widespread. AI is promoting the transformation of the healthcare system with unprecedented power. AI technology can enhance the work efficiency of healthcare personnel by identifying individual differences, assisting in early diagnosis, optimizing the diagnosis and treatment process, and so on [1]. Nursing, as the core link of medical services, is at the forefront of this technological revolution. The wide application of AI technologies such as electronic health record management, nursing robots, intelligent decision support systems, and digital telemedicine has made significant contributions to the improvement of nursing quality and efficiency [2, 3]. For example, Li et al. [4] utilized the AI‐health education accurately linking system to conduct physiological monitoring and regularly deliver individualized health education content for patients with nonspecific lower back pain to establish a reliable health management platform for the patients and effectively enhance their self‐management ability. Song et al.’s [5] research on the nursing workload of 7073 hospitalized patients across 72 wards in six hospitals of China showed that nursing robots had the potential to substitute for 62.37% of the total nursing workload per capita. Meanwhile, the continuous advancement of relevant policies worldwide (China’s promulgation of the “Opinions on Deeply Implementing the ‘Artificial Intelligence Plus’ Action” in 2025 [6] and the United States’ promulgation of the “Strategic Plan for the Application of Artificial Intelligence in Health, Public Services, and Public Health” [7]) indicates that the application of AI in the medical field will develop in a more in‐depth and standardized direction. As the executors and responsible persons of nursing work, nurses’ understanding and attitude toward AI technology have become the key factors determining whether AI can be deeply integrated and successfully applied in the field of nursing [8].

Nurses’ attitudes toward the use of AI referred to their cognitive evaluation and behavioral tendency toward the use of AI technology based on their perception and evaluation of AI technical resources in the working environment [9]. Existing research has been indicated that nurses’ AI attitudes exhibit significant complexity and contradiction. On the one hand, nurses generally recognized the benefits of AI and its development potential [10] and had expectations for its application in the field of nursing. On the other hand, nurses also showed anxiety about AI, namely, the negative emotional experiences such as tension, worry, and fear that individuals might have due to the development of AI technology and its potential impacts [11]. This emotion mainly stemmed from the fear of job replacement, inadaptability to the change of work mode, doubts about the reliability and accuracy of AI systems, and considerations about data privacy and ethical risks [10, 12, 13], which might lead to negative attitude toward the application of AI in nursing. Nurses’ AI attitudes have been confirmed to affect their willingness to use AI [14]. Therefore, it is particularly important to deeply explore the formation mechanism of nurses’ attitude toward the use of AI and formulate targeted strategies based on this.

The resource‐based theory revealed that the heterogeneity of organizational resources determined the differences in individual cognition, ability, and behavior [15]. China’s hospital service system is divided into three levels and 10 levels according to the Measures for Hierarchical Hospital Management, forming a hierarchical system of medical resources with hospital levels as the core, resulting in systematic differences in technical facilities, financial investment, talent structure, and strategic positioning of different medical institutions. As regional medical centers, tertiary hospitals possess a strong resource suction capacity [16], which can concentrate more advantageous resources and financial investment and have richer hardware resources, larger construction scale, more bed settings, and sophisticated equipment, so that nurses have more opportunities to contact and apply AI than those in secondary hospitals [17]. In contrast, the resources of secondary hospitals are relatively limited, and the pace of technology update is slow. This “digital resource gap” derived from the hospital hierarchy may lead to significant disparities in the cognition, evaluation, and use of AI, namely, “AI literacy” [18] of nurses in different levels of hospitals. AI literacy was defined as the comprehensive ability of individuals to understand, use, evaluate, and criticize AI technologies [19]. Higher AI literacy meant deeper understanding of AI technology, higher level of AI application skills, and more rigorous and objective thinking and attitude [20]. The social cognitive theory emphasized the interaction among the environment, individuals, and behaviors [21]. Studies have shown that the level of AI literacy can affect nurses’ anxiety level toward AI [22], and nurses’ AI anxiety level is closely related to their attitude toward AI [23, 24]. This complex mechanism is a deep reflection of the social cognition theory.

Although some progress has been made in the current research on the relationship among nurses’ AI literacy, anxiety, and attitude, the exploration of the mediating mechanism among the three still needs to be deepened. In addition, most of the existing studies focus on a single institution or a specific type of hospital, lacking a comparative perspective under different levels of hospital systems. It remains to be further explored whether and how hospital hierarchy differences affect nurses’ attitudes toward the use of AI through the chain‐mediated effect of AI literacy and anxiety level.

Based on this, this study, guided by the resource‐based theory and the social cognition theory, took nurses in Shandong, China, as the research subjects and constructed a theoretical model of “organizational resources‐individual cognition/emotion‐behavioral attitude” to explore the deep influence mechanism of hospital hierarchy on nurses’ attitudes toward AI. Based on the resource‐based theory, hospital grades, as an external manifestation of organizational resource heterogeneity, determined the differences in AI resources (training, equipment, and practical opportunities) that nurses obtained, which would directly affect nurses’ attitudes toward the use of AI. Combined with the social cognition theory, hospital hierarchy, as an environmental factor, indirectly affected nurses’ AI attitudes by changing their AI literacy and AI anxiety, and AI literacy could further influence AI anxiety, forming chain‐mediating effect. The two theories complemented each other and formed a logical loop, which not only explained the impact of organizational resource differences on individuals but also clarified the internal mechanism of individual cognition and emotion. Therefore, we proposed the following hypotheses:

 H1: hospital hierarchy is positively correlated with negative and positive attitudes toward the use of AI;
 H2a: hospital hierarchy (the higher the level) positively correlates negative attitude toward the use of AI through AI literacy;
 H2b: hospital hierarchy (the higher the level) positively correlates positive attitude toward the use of AI through AI literacy;
 H3a: hospital hierarchy (the higher the level) positively correlates negative attitude toward the use of AI through AI anxiety;
 H3b: hospital hierarchy (the higher the level) negatively correlates positive attitude toward the use of AI through AI anxiety;
 H4a: hospital hierarchy (the higher the level) positively correlates negative attitude toward the use of AI through the chain mediation path from AI literacy to anxiety;
 H4b: hospital hierarchy (the higher the level) negatively correlates positive attitude toward the use of AI through the chain mediation path from AI literacy to anxiety.

## 2. Methods

### 2.1. Study Design

This study employed a cross‐sectional survey design to conduct questionnaires on attitude toward the use of AI, AI literacy, and AI anxiety among nurses and explored the mediating effect of AI literacy and anxiety between hospital hierarchy differences and negative and positive attitudes toward the use of AI. Subjects were selected convenience sampling from 11 hospitals (5 tertiary hospitals and 6 secondary and below hospitals) in 5 cities in the eastern, central, and western parts of Shandong Province in November 2025. The minimum sample size of structural equation model was 200 cases [25], and the sample size was 10–15 times of the observed variables [26]. In this study, the observed variables were 19, and the sample size was 10 times of the number of variables. Considering 20% of invalid questionnaires, the sample size was calculated to be 238 cases. A priori power analysis was conducted using G∗Power 3.1. For multiple linear regression and structural equation models, an effect size of *f* ^2^ = 0.15 (moderate effect) was set, with *α* = 0.05 and the power 1 − *β* = 0.95. The results showed that the minimum sample size required was 204 cases. A total of 471 questionnaires were collected in this study, with 436 being valid. The effective recovery rate was 92.57%. Among them, the participation rate of tertiary hospitals was 89.88% and that of secondary and below hospitals was 95.79%.

### 2.2. Inclusion and Exclusion Criteria

Inclusion criteria were as follows: (1) those who have obtained the nurse qualification certificate and completed the registration; (2) those who did clinical nursing work for no less than 6 months; and (3) those who voluntarily participated in this study and signed the informed consent form. Exclusion criteria were as follows: (1) those who hold nonclinical positions and (2) those who temporarily leave their posts due to sick leave or maternity leave during the investigation period.

### 2.3. Instruments

#### 2.3.1. General Information Questionnaire

This study used a self‐designed questionnaire to collect general information of nurses, including gender, age, marital status, educational level, years of working experience, professional title, and hospital level; it also recorded whether they were specialized nurses, whether they had received AI training, and their understanding of AI.

#### 2.3.2. The Chinese Version of the Attitude Scale Toward the Use of AI Technologies in Nursing (ASUAITIN‐C)

The ASUAITIN was developed by Dilek Yilmaz et al. [9] in 2024 to evaluate clinical nurses’ attitudes toward the use of AI in nursing practice, consisting of two dimensions: negative attitudes (6 items) and positive attitudes (9 items). Each item was scored on a 5‐point Likert scale (1 = strongly disagree to 5 = strongly agree), all of which were positive scores. The total score ranged from 15 to 75. The higher the score on the negative dimension, the more negative the attitude; the higher the score on the positive dimension, the more positive the attitude. In the survey conducted by Hu et al. [27] on Chinese nurses, the overall Cronbach’s *α* coefficient for the scale was 0.785, with values of 0.920 and 0.948 for the negative and positive attitude dimensions.

#### 2.3.3. AI Literacy Scale (AILS)

The AILS developed by Wang et al. [28] was used to assess the AI literacy of the general population. It consisted of 12 items, including four dimensions: awareness, usage, evaluation, and ethics, with three items in each dimension. Awareness dimension referred to the ability to identify and comprehend AI technology during the use of AI applications. Usage dimension referred to the ability to apply AI technology to accomplish tasks proficiently. Evaluation dimension referred to the ability to analyze, select, and critically evaluate AI applications and their outcomes. Ethics dimension referred to the ability to be aware of the responsibilities and risks associated with the use of AI technology. Each item was scored on a 7‐point Likert scale (1 = strongly disagree to 7 = strongly agree), with the total score ranging from 12 to 84. The higher the score, the better the AI literacy. In the AI literacy survey conducted among Chinese nurses, the Cronbach’s *α* coefficient of the scale was 0.858 [18].

#### 2.3.4. AI Anxiety Scale (AIAS)

The AIAS was developed by Wang et al. [29] in 2019 to evaluate the anxiety level of AI in the general population. The scale consisted of 21 items, including four subdimensions: learning (8 items), job replacement (6 items), sociotechnical blindness (4 items), and AI configuration (3 items). Each item was scored on a 7‐point Likert scale (1 = strongly disagree to 7 = strongly agree), with the total score ranging from 21 to 147. The higher the score, the higher the anxiety level of the AI. The Cronbach’s *α* coefficient of this scale was 0.964, and its internal consistency was relatively high.

### 2.4. Data Collection

This study used the online electronic questionnaires to investigate. Before the survey, the purpose of the study, requirements, and privacy protection measures were explained to the subjects in detail, and voluntary participation was emphasized. After informed consent, the subjects completed the questionnaire independently. The questionnaire was set as mandatory questions to prevent omissions and ensure data integrity. Using anonymous survey methods, each person could only fill in the questionnaire once. Questionnaires with regular responses, a filling time of less than 120 seconds, and those that did not conform to logic were eliminated. Quality control of the filled questionnaires would be conducted at any time.

### 2.5. Statistical Analysis

The data were collated and analyzed by using SPSS 26.0 and AMOS 29.0. General demographic data were analyzed using descriptive statistical methods. *T*‐tests, ANOVA, and rank sum tests were used for comparisons between groups. Spearman correlation analysis was used to explore the correlations among the variables. The common method bias was tested using Harman single‐factor test and confirmatory factor analysis (CFA). If the fit of the single‐factor model was extremely poor, there was no serious common method bias. Multiple linear regression analysis was used to investigate the influencing factors of nurses’ negative and positive attitudes toward the use of AI. Variables with statistical significance from the single factor analysis were taken as independent variables, and then the collinearity (VIF < 10) was tested before entering the regression model. Model validation was conducted using CFA to verify the construct validity of each scale, reporting combined reliability (CR), average variance extracted value (AVE), and discriminant validity. The structural equation model was constructed using AMOS 29.0 software, and 5000 bootstrap repeated sampling was used. The 95% bias‐corrected confidence interval was used to test the mediating effect. If the 95% CI did not include 0, the mediating effect was significant. The test level *α* = 0.05.

### 2.6. Ethical Considerations

This study was approved by the Research Ethics Committee of Jinan Maternity and Child Care Hospital (approval number: KY R‐25‐096). The informed consent was obtained from all respondents before the investigation.

## 3. Results

### 3.1. Characteristics of the Sample

In the study, males accounted for 11.5% and females accounted for 88.5%. Most of the nurses were 18–30 years old (61.5%), were single (53.4%), held a bachelor’s degree (82.8%), had working experience ≤ 5 years (55.0%), held the professional title of “nurse” (67.9%), worked in tertiary hospitals (53.0%), were specialist nurses (57.8%), had experienced AI training (66.5%), and had a relatively good understanding of AI (34.6%). See Table 1 for details.

**TABLE 1:** General characteristics and comparison of the scores for negative and positive attitudes toward the use of AI (*n* = 436).

Variables	Category	*n* (%)	Negative attitude	Positive attitude	*t*/*F*/*U*	*P*
Gender	Male	50 (11.5)	16.42 ± 7.69	38.42 ± 5.04	−1.863①	0.062①
	Female	386 (88.5)	14.31 ± 6.46	38.19 ± 4.21	−1.568②	0.117②

Age	18–30	268 (61.5)	15.29 ± 6.94	38.12 ± 4.16	6.293①	0.043①
	31–45	156 (35.8)	13.33 ± 5.74	38.15 ± 4.61	6.649②	0.036②
	46–55	12 (2.8)	14.00 ± 8.57	41.08 ± 2.59		

Marital status	Single	233 (53.4)	14.30 ± 6.65	38.46 ± 3.82	1.020①	0.600①
	Married	201 (46.1)	14.86 ± 6.64	37.99 ± 4.72	3.236②	0.198②
	Widowed/divorced	2 (0.5)	13.00 ± 5.66	31.50 ± 10.61		

Educational status	Vocational high school	7 (1.6)	21.71 ± 4.96	37.14 ± 6.31	24.950①	< 0.001①
	College	48 (11.0)	18.31 ± 6.56	36.48 ± 5.44	4.586②	0.205②
	Bachelor’s degree	361 (82.8)	13.98 ± 6.52	38.48 ± 4.01		
	Master’s degree or above	20 (4.6)	13.30 ± 4.98	37.95 ± 4.99		

Years of work experience	≤ 5	240 (55.0)	14.50 ± 6.69	38.32 ± 4.03	4.900①	0.298①
	6–10	132 (30.3)	13.99 ± 6.51	38.38 ± 4.31	7.169②	0.127②
	11–15	42 (9.6)	16.10 ± 6.66	37.02 ± 5.64		
	16–20	14 (3.2)	15.64 ± 5.61	37.29 ± 4.14		
	> 20	8 (1.8)	15.25 ± 8.48	40.13 ± 4.52		

Professional title	Nurse	296 (67.9)	14.43 ± 6.82	38.68 ± 3.55	9.221①	0.056①
	Nurse practitioner	75 (17.2)	14.72 ± 6.51	37.99 ± 4.33	11.569②	0.021②
	Charge nurse	56 (12.8)	14.16 ± 5.34	36.38 ± 6.56		
	Deputy chief nurse	4 (0.9)	15.00 ± 8.72	37.50 ± 3.70		
	Chief nurse	5 (1.1)	23.40 ± 4.83	35.20 ± 8.08		

Hospital hierarchy	Tertiary	231 (53.0)	16.75 ± 7.43	38.41 ± 4.21	−6.450①	< 0.001①
	Secondary and lower	205 (47.0)	12.07 ± 4.47	37.99 ± 3.43	−2.734②	0.006②

Specialist nurses	Yes	252 (57.8)	16.97 ± 7.23	38.12 ± 4.40	−8.032①	< 0.001①
	No	184 (42.2)	11.24 ± 3.71	38.35 ± 4.20	−0.305②	0.760②

AI training	Yes	290 (66.5)	13.05 ± 6.25	38.97 ± 4.10	−7.473①	< 0.001①
	No	146 (33.5)	17.54 ± 6.37	36.72 ± 4.34	−7.668②	< 0.001②

AI understanding level	No understanding	25 (5.7)	16.56 ± 5.40	35.72 ± 5.22	77.786①	< 0.001①
	General understanding	149 (34.2)	17.21 ± 6.13	36.23 ± 5.43	84.716②	< 0.001②
	Relatively good understanding	151 (34.6)	14.05 ± 6.86	39.28 ± 2.83		
	Very good understanding	111 (25.5)	11.22 ± 5.56	39.99 ± 2.31		

*Note:* ① comparison of the scores for negative attitude; ② comparison of the scores for positive attitude.

### 3.2. Nurses’ Negative and Positive Attitudes Toward the Use of AI, AILS and AIAS Scores, and Correlation Analysis

The score of negative attitude was 14.55 ± 6.63, the score of positive attitude was 38.21 ± 3.87, the score of AILS was 60.32 ± 6.10, and the score of AIAS was 73.67 ± 32.62 (see Table 2 for details). Spearman correlation analysis showed that AI literacy was positively correlated with the negative and positive attitudes toward the use of AI (*r* = 0.095, 0.249,*p* < 0.01). AIAS was strongly positively correlated with negative attitude (*r* = 0.775, *p* < 0.01) and weakly negatively correlated with positive attitude (*r* = −0.328, *p* < 0.01) (see Table 3 for details).

**TABLE 2:** Nurses’ negative and positive attitudes toward the use of AI and AILS and AIAS scores.

Variables	M ± SD	Tertiary hospitals	Secondary and lower hospitals
Negative attitude	14.55 ± 6.63	16.75 ± 7.43	12.07 ± 4.47
Positive attitude	38.21 ± 3.87	38.41 ± 4.21	37.99 ± 3.43
AILS	60.32 ± 6.10	61.48 ± 6.32	59.00 ± 5.57
Awareness	14.37 ± 2.02	14.74 ± 2.19	13.95 ± 1.72
Usage	15.00 ± 2.41	15.71 ± 2.60	14.19 ± 1.87
Evaluation	17.22 ± 2.47	17.16 ± 2.30	17.28 ± 2.65
Ethics	13.73 ± 2.03	13.87 ± 2.30	13.58 ± 1.67
AIAS	73.67 ± 32.62	85.17 ± 32.31	60.71 ± 28.82
Learning	27.84 ± 14.15	32.61 ± 15.21	22.48 ± 10.56
Job replacement	21.87 ± 9.96	25.30 ± 9.49	18.00 ± 9.04
Sociotechnical blindness	14.42 ± 6.68	16.63 ± 6.52	11.92 ± 5.94
AI configuration	9.54 ± 4.85	10.64 ± 5.37	8.31 ± 3.84

*Note:* M, mean.

Abbreviations: AIAS, artificial intelligence anxiety scale; AILS, artificial intelligence literacy scale; SD, standard deviation.

**TABLE 3:** Correlation analysis of nurses’ negative and positive attitudes toward the use of AI with AILS and AIAS.

Variables	Negative attitude	Positive attitude
AILS	0.095^∗^	0.249^∗∗^
Awareness	0.163^∗∗^	0.002
Usage	0.280^∗∗^	0.149^∗∗^
Evaluation	−0.386^∗∗^	0.519^∗∗^
Ethics	0.096^∗^	−0.012
AIAS	0.775^∗∗^	−0.328^∗∗^
Learning	0.741^∗∗^	−0.287^∗∗^
Job replacement	0.683^∗∗^	−0.355^∗∗^
Sociotechnical blindness	0.680^∗∗^	−0.314^∗∗^
AI configuration	0.643^∗∗^	−0.344^∗∗^

Abbreviations: AIAS, artificial intelligence anxiety scale; AILS, artificial intelligence literacy scale.

^∗^ *p* < 0.05.

^∗∗^ *p* < 0.01.

### 3.3. Common Method Bias Test

The Harman single‐factor test showed that the variance interpretation rate of the first common factor extracted for all items was 28.06%, which was less than the critical value of 40%. The fitting results of the CFA single‐factor model were *χ* ^2^/df = 16.938, RMSEA = 0.191, CFI = 0.751, and TLI = 0.688. The fit was poor, indicating that there was no serious common method bias in this study.

### 3.4. Nurses’ Negative and Positive Attitudes Toward the Use of AI Influencing Factors

The single factor analysis results showed that age, educational status, hospital hierarchy, specialist nurses, AI training, and understanding of AI had statistically significant effects on nurses’ negative attitude toward the use of AI (*p* < 0.05). Age, professional title, hospital hierarchy, AI training, and understanding of AI had statistically significant effects on nurses’ positive attitude toward the use of AI (*p* < 0.05). See Table 1 for details. Taking the score of nurses’ negative and positive attitudes toward the use of AI as the dependent variable and the variables with statistical significance in the single factor analysis as the independent variables, a multiple linear regression analysis was conducted. Before the multiple linear regression, the variables were first assigned and dummy variables were set in order to explain the results. The collinearity diagnosis showed that the VIF of all independent variables were < 10, indicating that there was no severe collinearity. The analysis showed that hospital hierarchy, AI literacy, and AI anxiety were the influencing factors of nurses’ negative attitude toward the use of AI (*p* < 0.05). Age (46–55 years), professional title (charge nurse and chief nurse), hospital hierarchy, understanding of AI (relatively good understanding and very good understanding), AI literacy, and AI anxiety had statistically significant effects on nurses’ positive attitude toward the use of AI (*p* < 0.05). See Tables 4, 5, and 6 for details.

**TABLE 4:** Independent variable and dummy variable assignment.

Independent variables	Assignment
Age	18–30 = 0; 31–45 = (0, 1, 0); 46–55 = (0, 0, 1)
Educational status	Vocational high school = 0; college = (0, 1, 0, 0); bachelor’s degree = (0, 0, 1, 0); master’s degree or above = (0, 0, 0, 1)
Professional title	Nurse = 0; nurse practitioner = (0, 1, 0, 0, 0); charge nurse = (0, 0, 1, 0, 0); deputy chief nurse = (0, 0, 0, 1.0); chief nurse= (0, 0, 0, 0, 1)
Hospital hierarchy	0 = secondary and lower; 1 = tertiary
Specialist nurses	0 = no; 1 = yes
AI training	0 = no; 1 = yes
AI understanding	No understanding = 0; general understanding= (0, 1, 0, 0); relatively good understanding= (0, 0, 1, 0); very good understanding= (0, 0, 0, 1)
AILS	Substitute the original value
AIAS	Substitute the original value

Abbreviations: AIAS, artificial intelligence anxiety scale; AILS, artificial intelligence literacy scale.

**TABLE 5:** Multivariate analysis of influencing factors of nurses’ negative attitude toward the use of AI.

Variables	*B*	SE	*β*	*t*	*P*
Constant	10.478	3.041		3.446	0.001
Age					
18–30 (reference)					
31–45	0.055	0.435	0.004	0.126	0.900
46–55	1.890	1.224	0.047	1.544	0.123
Educational status					
Vocational high school (reference)					
College	0.165	1.705	0.008	0.097	0.923
Bachelor’s degree	−1.044	1.627	−0.059	−0.642	0.521
Master’s degree or above	−0.962	1.867	−0.030	−0.516	0.606
Hospital hierarchy					
Secondary and lower (reference)					
Tertiary	1.102	0.456	0.083	2.415	0.016
Specialist nurses					
No (reference)					
Yes	0.839	0.507	0.063	1.655	0.099
AI training					
No (reference)					
Yes	−0.109	0.664	−0.008	−0.165	0.869
AI understanding level					
No understanding (reference)					
General understanding	−0.206	0.941	−0.015	−0.219	0.827
Relatively good understanding	−0.250	1.056	−0.018	−0.236	0.813
Very good understanding	1.352	1.139	0.089	1.187	0.236
AILS	0.070	0.033	0.064	2.096	0.037
AIAS	0.141	0.008	0.692	17.272	< 0.001

*Note:* *R* ^2^ = 0.640; adjusted *R* ^2^ = 0.629; *F* = 57.811; *p* < 0.001; DW = 1.514. *B*, unstandardized beta; *F*, model statistics; *P*, level of significance; *R*, correlation; *R* ^2^, correlation coefficient (explained variance ratio); *β*, standardized beta.

Abbreviations: AIAS, artificial intelligence anxiety scale; AILS, artificial intelligence literacy scale; SE, standard error.

**TABLE 6:** Multivariate analysis of influencing factors of nurses’ positive attitude toward the use of AI.

Variables	*B*	SE	*β*	*t*	*P*
Constant	28.305	1.979		14.299	< 0.001
Age					
18–30 (reference)					
31–45	0.567	0.452	0.063	1.256	0.210
46–55	4.349	1.195	0.165	3.639	< 0.001
Professional title					
Nurse (reference)					
Nurse practitioner	−0.417	0.511	−0.037	−0.817	0.414
Charge nurse	−1.729	0.675	−0.134	−2.560	0.011
Deputy chief nurse	−2.886	1.893	−0.064	−1.524	0.128
Chief nurse	−3.939	1.753	−0.097	−2.247	0.025
Hospital hierarchy					
Secondary and lower (reference)					
Tertiary	1.814	0.408	0.210	4.449	< 0.001
AI training					
No (reference)					
Yes	0.148	0.516	0.016	0.287	0.774
AI understanding level					
No understanding (reference)					
General understanding	−0.108	0.835	−0.012	−0.130	0.897
Relatively good understanding	1.957	0.930	0.216	2.105	0.036
Very good understanding	2.166	1.000	0.219	2.167	0.031
AILS	0.174	0.032	0.247	5.381	< 0.001
AIAS	−0.038	0.007	−0.289	−5.864	< 0.001

*Note:* *R* ^2^ = 0.314; adjusted *R* ^2^ = 0.312; *F* = 14.836; *p* < 0.001; DW = 1.904. *B*, unstandardized beta; *F*, model statistics; *P*, level of significance; *R*, correlation; *R* ^2^, correlation coefficient (explained variance ratio); *β*, standardized beta.

Abbreviations: AIAS, artificial intelligence anxiety scale; AILS, artificial intelligence literacy scale; SE, standard error.

### 3.5. Mediating Effect Analysis of AI Literacy and Anxiety Between Hospital Hierarchy Differences and Negative and Positive Attitudes Toward the Use of AI

The results of CFA showed that the four‐factor model provided the best fit compared to other models. The results of model fit were *χ* ^2^/df = 3.169, GFI = 0.974, AGFI = 0.918, NFI = 0.977, IFI = 0.984, TLI = 0.958, CFI = 0.984, and RMSEA = 0.071. As summarized in Table 7, introducing a common method variability (CMV) factor resulted in negligible changes in model fit indices when comparing the five‐factor model with the four‐factor model. Specifically, the differences in RMSEA and SRMR were less than 0.05, and increases in CFI and TLI were less than 0.01. These findings provided strong evidence that common method bias did not significantly affect the validity of this study.

**TABLE 7:** Fit statistics derived from confirmatory factor analysis.

Measurement model	*χ* ^2^/df	RMSEA	SRMR	CFI	TLI
Single factor (HH + AILS + AIAS + ASUAITIN)	16.938	0.191	0.125	0.751	0.688
Two factors (HH + ASUAITIN, AILS + AIAS)	12.688	0.164	0.110	0.830	0.771
Three factors (HH, AILS + AIAS, ASUAITIN)	7.996	0.127	0.078	0.915	0.863
Four factors (HH, AILS, AIAS, ASUAITIN)	3.169	0.071	0.054	0.984	0.958
Five factors (HH, AILS, AIAS, ASUAITIN, CMV)	2.964	0.067	0.043	0.985	0.962

*Note:* ASUAITIN, attitude scale toward the use of artificial intelligence technologies in nursing.

Abbreviations: AIAS, artificial intelligence anxiety scale; AILS, artificial intelligence literacy scale; CMV, common method variability; HH, hospital hierarchy.

**FIGURE 1:**
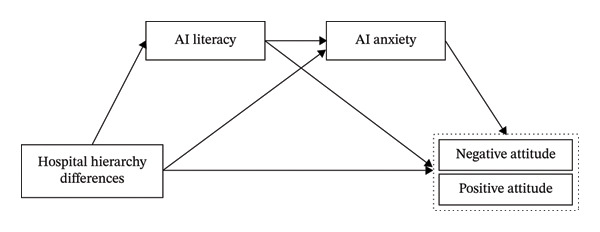
Mediation model diagram of nurses’ AI literacy and anxiety between hospital hierarchy differences and negative and positive attitudes toward the use of AI.

In the mediation model, hospital hierarchy was the independent variable, AI literacy and anxiety were the mediating variables, and negative and positive attitudes toward the use of AI were the dependent variables. As shown in Figure 1, CFA was used to verify the construct validity of each scale. The results showed that CR of each dimension was all > 0.7, and AVE was all > 0.5, indicating that the scales had good convergent validity; the square root of AVE of each dimension was all greater than its correlation coefficients with other dimensions, indicating that the scales had good discriminant validity and there was no problem of concept overlap. The analysis showed that hospital hierarchy had a direct effect on negative and positive attitudes toward the use of AI, with effect sizes of 1.322 and 1.836, respectively (*p* < 0.05). The indirect effects of AI literacy and anxiety between hospital hierarchy differences and negative and positive attitudes toward the use of AI, included six mediating paths: (1) mediating path 1 composed of hospital hierarchy‐AI literacy‐negative attitude toward the use of AI, with effect size of 0.202; (2) mediating path 2 composed of hospital hierarchy‐AI anxiety‐negative attitude toward the use of AI, with effect size of 0.258; (3) mediating path 3 composed of hospital hierarchy‐AI literacy‐AI anxiety‐negative attitude toward the use of AI, with effect size of 0.285; (4) mediating path 4 composed of hospital hierarchy‐AI literacy‐positive attitude toward the use of AI, with effect size of 0.446; (5) mediating path 5 composed of hospital hierarchy‐AI anxiety‐positive attitude toward the use of AI, with effect size of −1.197; (6) mediating path 6 composed of hospital hierarchy‐AI literacy‐AI anxiety‐positive attitude toward the use of AI, with effect size of −0.260 (95% CI did not include 0, *p* < 0.05). See Tables 8 and 9 for details.

**TABLE 8:** Chain mediating effect analysis of nurses’ AI literacy and anxiety between hospital hierarchy differences and negative attitude.

Path	Estimate	SE	95% CI	*P*
Direct effect	1.322	0.473	0.410–2.272	0.004
Total indirect effect	3.067	0.442	2.185–3.906	0.001
Path 1	0.202	0.099	0.067–0.429	0.003
Path 2	2.580	0.430	1.788–3.508	< 0.001
Path 3	0.285	0.138	0.112–0.583	0.001
Total effect	4.389	0.555	3.328–5.473	< 0.001

Abbreviations: CI, confidence interval; SE, standard error.

**TABLE 9:** Chain‐mediating effect analysis of nurses’ AI literacy and anxiety between hospital hierarchy differences and positive attitude.

Path	Estimate	SE	95% CI	*P*
Direct effect	1.836	0.414	1.060–2.661	< 0.001
Total indirect effect	−1.011	0.324	−1.677∼−0.382	0.002
Path 4	0.446	0.242	0.029–1.008	0.038
Path 5	−1.197	0.265	−1.775∼−0.718	< 0.001
Path 6	−0.260	0.144	−0.594∼−0.014	0.039
Total effect	0.825	0.381	0.081–1.582	0.029

Abbreviations: CI, confidence interval; SE, standard error.

## 4. Discussion

This study aimed to explore the impact of hospital hierarchy differences on negative and positive attitudes toward the use of AI in the nursing field with AI literacy and anxiety as mediating variables. The results showed that hospital hierarchy differences were significantly correlated with attitude toward the use of AI. Meanwhile, hospital hierarchy differences could affect attitude toward the use of AI through the following channels: (1) hospital hierarchy differences ⟶ AI literacy ⟶ negative and positive attitudes toward the use of AI; (2) hospital hierarchy differences ⟶ AI anxiety ⟶ negative and positive attitudes toward the use of AI; (3) hospital hierarchy differences ⟶ AI literacy ⟶ AI anxiety ⟶ negative and positive attitudes toward the use of AI. The results deepen our understanding of the mechanism of action of these four variables and provided theoretical basis for promoting the application of AI in the field of nursing.

### 4.1. Analysis of the Current Situation and Influencing Factors of Clinical Nurses’ Negative and Positive Attitudes Toward the Use of AI

The results of this study showed that the score of clinical nurses’ negative attitude toward the use of AI was 14.55 ± 6.63 and the score of nurses’ positive attitude toward the use of AI was 38.21 ± 3.87, which was similar to the research results of Hu et al. [27]. The regression analysis showed that the factors affecting negative and positive attitudes toward the use of AI were different. Specifically, the influencing factors of negative attitude were relatively single, mainly related to the hospital level. The influencing factors of positive attitude were multiple, including age (46–55 years), professional title (charge nurse and chief nurse), understanding of AI (relatively good understanding and very good understanding), and hospital level. The negative and positive attitudes of nurses in tertiary hospitals were higher than those in secondary and below hospitals, which was consistent with the research results of Zeng et al. [30]. This difference might be related to the inherent systemic factors of the medical hierarchical structure. Tertiary hospitals held the dominant position in medical services, with leading medical technologies and facilities [31]. From AI‐assisted diagnosis to intelligent patient monitoring systems, nurses might be frequently contacted with various AI tools in their work. On the one hand, direct practical experience could enable nurses to specifically understand the actual utility of AI applications, forming an effective sense of technical value recognition and thus might have a more positive attitude toward the use of AI. On the other hand, repeated operational practices might also enable nurses to master the use of AI technology proficiently, thereby possibly generating a better sense of AI self‐efficacy, which was closely related to positive attitude toward the use of AI [30, 32]. Nurses in tertiary hospitals had a higher negative attitude toward the use of AI than those in secondary and lower hospitals, which might be due to different work environments and pressures (when dealing with more complex patients, nurses might tend to trust their own experience and judgment more than the standardized suggestions of AI), diverse career development directions of individuals, different decision‐making methods of management, and the technical black box [33]. Nurses aged 46 to 55 scored higher in a positive attitude toward the use of AI, which might be related to the fact that nurses in this age group were in the mature stage of their career development and held open attitude toward new technologies. Khan et al. [34] showed that medical staff in the 18–25 and 26–35 age groups might have a more positive attitude toward AI, which was different from the results of this study. This difference might be attributed to differences in countries and regions, research subjects, or AI application scenarios, suggesting that the influence of age on the attitude toward the use of AI might vary in different situations. However, the sample size for this age group in this study was relatively small (*n* = 12), and this finding needs to be verified by expanding the sample size. In terms of professional titles, regression analysis showed that only the charge nurse and the chief nurse had statistically significant impact on the positive attitude toward the use of AI and was negatively correlated. The reason might be that certain high‐ranking nurses might have a more conservative attitude toward AI technology and tend to adopt traditional working methods. However, the sample size of chief nurses was only 5, and the robustness of this result needs further verification. In terms of understanding of AI, regression analysis showed that compared with nurses who had no understanding of AI, those who had relatively good understanding and very good understanding of AI had better positive attitude toward the use of AI, and the impact was statistically significant. This might be because nurses who had relatively good understanding of AI had lower sense of uncertainty and unfamiliarity with AI, and thus might be more willing to operate and practice it when they came into contact with AI. Based on the above findings, it is recommended that managers implement personalized management for nurses based on their individual differences. In response to negative attitudes, particular attention should be paid to the risk perception and emotional counseling of nurses in tertiary hospitals. In response to positive attitudes, it would be necessary to enhance the knowledge training for nurses with relatively low subjective cognitive evaluations of AI, attach importance to the attitude guidance for charge nurses and chief nurses, and at the same time, strengthen the positive leading role of middle‐aged backbone nurses aged 46 to 55. The allocation of AI resources in hospitals at different levels should be balanced to promote the popularization and application of AI technology in the field of nursing.

### 4.2. Mediating Role of AI Literacy Between Hospital Hierarchy Differences and Nurses’ Negative and Positive Attitudes Toward the Use of AI

The mediation analysis of this study showed that hospital hierarchy differences were positively correlated with nurses’ negative attitude toward the use of AI through the mediating effect of AI literacy, with the effect value of 0.202, and were positively correlated with positive attitude, with the effect value of 0.446. Previous studies had shown [23, 30] that there was a significant positive correlation between AI literacy and nurses’ attitudes toward the use of AI. The AI literacy of nurses in tertiary hospitals was significantly higher than that of nurses in secondary and below hospitals. This might be related to differences in working environment and resources. On the one hand, tertiary hospitals had better material foundation and working platform, which might enable nurses to have more opportunities to contact with advanced and new AI technologies [30]. Meanwhile, structured training might enable nurses to receive systematic and standardized knowledge. On the other hand, tertiary hospitals should gather nursing talents with high education and strong learning ability and have relatively strong research atmosphere. The above factors might jointly promote a relatively favorable environment for improving AI literacy, which might help enhance nurses’ abilities in understanding, applying, and critically evaluating AI technologies, promote their profound understanding of the advantages and disadvantages of AI, and possibly promote the positive association with negative and positive attitude toward the use of AI. While secondary and lower‐level hospitals might have issues such as limited resources, weak work demands, and conservative management attitudes, nurses might have a low level of AI literacy, might be accompanied by cognitive misunderstanding and emotional estrangement toward AI, which might result in both low scores for negative and positive attitudes toward the use of AI. This suggested that the level of AI literacy may be positively correlated with both types of attitudes, but this correlation was relatively weak in secondary and lower‐level hospitals. Varnosfaderani et al. [35] pointed out that hospitals with strong and advanced technological infrastructure were more likely to promote the application of AI in clinical settings. Therefore, when promoting the development of AI in the nursing field, it is recommended to implement the hierarchical AI training strategy. For tertiary hospitals, advanced AI application and critical assessment training could be carried out. For secondary and lower‐level hospitals, policy support could be provided to enhance nurses’ basic understanding and practical experience in AI, helping them bridge the digital divide.

### 4.3. Mediating Role of AI Anxiety Between Hospital Hierarchy Differences and Nurses’ Negative and Positive Attitudes Toward the Use of AI

The results of this study showed the mediating role of AI anxiety between hospital hierarchy differences and nurses’ negative and positive attitudes toward the use of AI, with the effect value of 2.580 and −1.197, respectively, making it the core path with the highest proportion among all paths, suggesting that hospital hierarchy differences among nurses might have positive correlation with negative attitude toward the use of AI and negative correlation with positive attitude toward the use of AI through AI anxiety. This study showed that AI anxiety was strongly positively correlated with negative attitude and weakly negatively correlated with positive attitude, which was consistent with previous studies [36]. Tertiary hospitals had complex work environment, high work intensity, and fast work pace; nurses might need to handle high‐load patient care, complex disease changes, and continuous quality assessments in their daily work. Under this high‐pressure mode, the introduced AI technology might become new source of stress. Nurses could be likely to have technical adaptation anxiety caused by work content changes, responsibility ownership anxiety caused by judging AI decisions, and ethical safety anxiety caused by processing patient privacy data when accepting new AI technology [37, 38]. These anxieties might become more prominent due to rapid technological advancements and the deepening of application scenarios and might thus be associated with relatively low positive attitude toward the use of AI. However, due to the slow introduction of new technologies and the regularity of work in secondary and lower‐level hospitals, nurses might have a relatively vague perception of AI, and their AI anxiety level might also be relatively low. These nurses might not have deeply engaged in AI‐assisted decision‐making practices, and their concerns about AI might include job substitution anxiety, learning anxiety, and technological blindness anxiety [39]. These survival anxieties might also reinforce negative attitude by threatening job security. The study had shown when nurses perceived that AI technology might replace their own positions, AI anxiety might significantly inhibit the willingness for human‐machine cooperation, manifested as avoidance, resistance, and distrust toward the application of technology, which might be related to more negative attitude toward the use of AI [39]. Therefore, whether it was the developmental anxiety of tertiary hospitals or the survival anxiety of secondary hospitals, it was all a negative psychological experience. This finding supported a possible psychological pathway: AI anxiety might play a mediating role between nurses’ cognitive assessment of AI and their behavioral intentions, and it showed opposite relationship directions for negative and positive attitudes; it was positively correlated with negative attitude and negatively correlated with positive attitude. It suggested that nursing managers could take AI anxiety intervention as a key task and focus on differentiating anxiety types and implementing targeted emotional counseling.

### 4.4. Chain Mediating Effect of AI Literacy and Anxiety Between Hospital Hierarchy Differences and Nurses’ Negative and Positive Attitudes Toward the Use of AI

In addition, this study observed a chain mediating path: hospital hierarchy ⟶ AI literacy ⟶ AI anxiety ⟶ negative and positive attitudes toward the use of AI, with the effect value of 0.285 and −0.260, respectively. Although the proportion was not large, it might suggest the possible correlation between cognitive factors and emotional factors. The resource differences caused by hospital hierarchy differences may, to some extent, affect the AI literacy of nurses. The differences in AI cognition and understanding could lead to varying degrees of AI anxiety [22]. This anxiety showed a certain corresponding relationship with nurses’ negative and positive attitudes toward the use of AI [24]. Specifically, tertiary hospitals might be inclined to introduce and promote AI technology because of their strategic positioning, which might provide a lot of learning and practice opportunities for nurses. Nurses who demonstrated a high level of AI literacy might have deeper understanding and insight of AI, recognizing its application value and potential risks, which might manifest specific and relatively high level of AI anxiety. Anxiety might activate an individual’s defensive psychological system, causing them to be more likely to focus on the potential risks (such as liability for misdiagnosis and privacy leakage) of technology rather than its potential benefits. This risk perception bias might be accompanied by lower willingness to use AI and positive evaluations [40]. Meanwhile, individuals with anxiety might be prone to “technological resistance”, manifested as distrust of AI, concerns about the consequences of its use, and attachment to traditional working methods. These tendencies might be associated with negative attitude toward the use of AI. This result suggested a hypothetical intervention approach. Before the introduction of AI technology, AI literacy training should be given priority to improve the cognitive level of nurses, relieve their anxiety, and at the same time, promote the formation of their positive AI use attitude, while appropriately addressing their negative attitude toward AI.

## 5. Precision Intervention Strategies for Nursing Management

Based on the results of this study, considering the heterogeneity of resources in different levels of hospitals and the individual characteristics of the nursing staff, it would develop stratified, precise, and operational nursing management intervention measures to promote the deep integration of AI in the field of nursing.

### 5.1. Tertiary Hospitals: Deepen AI Application and Focus on Anxiety Intervention and Human–Machine Collaboration

Nursing staff in tertiary hospitals generally had anxiety related to their professional development. It would be necessary to focus on strengthening the emotional counseling and psychological support for nurses, optimizing the human–machine collaboration work system, and fully leveraging the clinical application value of AI. First, it would establish a clear psychological support system and human–machine collaboration system to regularly conduct psychological assessments for nurses in the hospital, identify the attributes of their anxiety, and adopt corresponding strategies. For example, anxiety caused by unclear responsibility definition could be addressed by formulating a complete human–machine collaboration process guide and responsibility division details, clearly defining the supervisory responsibilities and exemption boundaries of nurses to reduce their perception of occupational risks; ethical security anxiety could be addressed by conducting AI data privacy protection training, standardizing the data collection and application process of AI, and protecting patient privacy and the occupational safety of nurses [41]. Secondly, efforts should be made to promote clinical empowerment and interdisciplinary collaboration. Based on the characteristics of various operating rooms, ICU, and other specialized departments, advanced application training such as AI intelligent decision support systems and specialized nursing robots should be introduced for training to enhance nurses’ ability to innovate with AI [42]. Relying on the hospital’s research platform, nursing research groups should be established to encourage nurses to participate in scenario‐based testing of AI nursing research and to reduce the problem of technical black boxes and ensure that the developed technologies would be suitable for clinical application scenarios and meet the needs of frontline nursing. Thirdly, the evaluation system and incentive mechanism should be improved. The application ability of AI should be included in the comprehensive assessment criteria for nursing staff. A new dimension of “innovative application of nursing technology” should be added to the evaluation of professional titles. Nurses who produce high‐quality AI application cases, participate in research and development, or propose effective improvement plans would be recognized. Through institutionalized positive feedback, the external pressure of technological change could be transformed into the internal growth motivation of individual nurses.

### 5.2. Secondary and Below‐Level Hospitals: Promoting AI Application and Focus on AI Basic Training and Resource Assistance

Nurses in secondary and below‐level hospitals had issues such as low AI literacy and survival anxiety, which could be alleviated by popularizing AI knowledge and increasing resource investment. Firstly, a step‐by‐step training system of “AI basic understanding‐practical application‐simple assessment” could be established to gradually enhance nurses’ understanding and application ability of AI [43]. In the first stage, the basic concepts of AI and its common application scenarios in the nursing field would be popularized through online classes and short videos. In the second stage, practical training on the use of intelligent vital sign monitors and AI‐assisted report interpretation would be conducted mainly in the hospital environment. In the third stage, nurses would be encouraged to conduct simple evaluations of AI applications in their daily work to cultivate their evidence‐based thinking. Secondly, through case sharing and on‐site training methods, nurses could gain a clear understanding of the practical value of AI in improving nursing efficiency and reducing workloads, thereby reducing their concerns about job substitution and transforming their survival anxiety into learning motivation. Finally, hospitals should actively seek policy and resource support from local health administrative departments [44] and provide basic AI nursing equipment to clinical departments, such as electronic health record management systems and intelligent vital sign monitors. At the same time, the targeted assistance mechanism for AI technology from tertiary hospitals to secondary and lower‐level hospitals should be established to promote the sharing of high‐quality AI resources and make up for the shortcomings in AI application in grassroots hospitals.

### 5.3. Improving the AI Nursing Management System and Ensuring the Standardized Application of AI

Regular monitoring of nurses’ AI literacy and anxiety should be established and incorporated into the nurses’ professional ability assessment system. Problems should be identified and intervened in a timely manner through regular monitoring. Relevant industry norms for AI nursing should be formulated to clearly define the application scenarios, operation procedures, and responsibility divisions of AI in the field of nursing, so that nurses have rules to follow and laws to abide by. AI‐related courses should be integrated into nursing school education and continuing education to strengthen the cultivation of comprehensive AI nursing talents, thereby providing sustainable human resources support for the intelligent transformation of the nursing discipline.

## 6. Conclusion

Hospital hierarchy differences could directly positively predict the negative and positive attitudes toward the use of AI and could also positively correlate the negative attitude toward the use of AI through mediation by AI literacy and anxiety and negatively correlate the positive attitude toward the use of AI. Nursing managers should fully consider the resource differences at various hospital levels and formulate stratified and precise educational and training intervention strategies related to AI to improve nurses’ understanding of AI and enhance their AI literacy. At the same time, nursing managers should monitor the emotional state of nurses, regularly assess their mental health, and take supportive measures to alleviate their AI anxiety. Furthermore, incorporating AI technology into state‐controlled medical policies and ensuring the safety and reliability of software and hardware will help change nurses’ acceptance of AI and thereby enhance their attitude toward using it.

## 7. Limitations

This study was the first to deeply explore the role of the hospital hierarchy of clinical nurses in China on attitude toward the use of AI through AI literacy and anxiety, which provided support for hospitals of different levels to promote the application of AI in clinical nursing work and promote human–machine collaboration in the nursing industry. This study had several limitations. Firstly, the research data originated from cross‐sectional surveys, making it impossible to verify causal relationships. The sample size included was relatively small, with potential selection bias, and the generalization of the research results might be restricted, etc. Secondly, all the tools used for data collection in this study were all self‐reported scales, which inherently carried a degree of subjectivity and might lead to biased results. Additionally, factors such as self‐efficacy, perceived substitution, and digital leadership, which might influence attitude toward the use of AI, were not considered. Future research could conduct multicenter and large‐sample investigation studies to enhance the universality of the results. It would be also necessary to combine qualitative research and longitudinal study methods to more deeply explore the factors influencing clinical nurses’ attitudes toward the use of AI, providing references for formulating targeted intervention measures. In addition, it would be necessary to explore the mediating and moderating factors of self‐efficacy, perceived substitution, and digital leadership in order to enhance comprehensive understanding of nurses’ attitudes toward the use of AI.

## Author Contributions

All authors contributed to the study design. Yimeng Zheng drafted, revised, and reviewed the manuscript. Ding Xu, Yimeng Zheng, Linlin Yang, Beibei Liang, and Jielong Du contributed to the data collection. Ding Xu conducted the data analysis and revised and reviewed the manuscript. Yongtian Yin was involved in the study supervision. Cuixia Lin provided financial support and supervised the study. All authors contributed to the manuscript preparation. Ding Xu, Yimeng Zheng, Yongtian Yin, and Linlin Yang are the co‐first authors. Cuixia Lin is the corresponding author, E‐mail: breezelinlin@163.com.

## Funding

No funding was received for this research.

## Disclosure

All authors reviewed and approved the final draft of the manuscript.

## Conflicts of Interest

The authors declare no conflicts of interest.

## Data Availability

The data that support the findings of this study are available on request from the corresponding author. The data are not publicly available due to privacy or ethical restrictions.
